# Supplementary material for: Chromothripsis during telomere crisis is independent of NHEJ, and consistent with a replicative origin
Source: Genome Res. 2019 May;29(5):737–49. doi: 10.1101/gr.240705.118 (PMC6499312; doi:10.1101/gr.240705.118)
Supplement: Supplemental Material [file supp_gr.240705.118_Supplemental_file_1.zip › contigs/annotated_contigs/DB38/contig.2.DB38_length_865_mean_cov_8.56300578035.docx]

**DB38_length_865_mean_cov_8.56300578035**

```
ATATATACACACACACATCTATATCTATCTATATATCTATACATATATATACACACACACAATGTAATAAGAAAATTCTGATACATGCT
 >chr3:188318379-188318763 - E=4e-219
ACAACATAGATGAAGCTTGAAGAGATTAAGCTTGAAGCTTGAAGAAACAAGCCAGTTACAAAAACAAAAAACAAAAAACAAATACCATA

TGGTTCTACATATAGGAGGCCCCTAGAAGAGTCAAATTTATAAAGCTAGAAAGCAGAATGGTGATTGCCATGGGATGGGAGAAGGAAGA

ATGTAGCTAGTGCATTATTGTTTAATGGATATGTCATTTCAGTTTGAATAGATACAGTTCTGCTGGTGATGGTTGCACAACAATGTAAA

TGTACTTAGTGCCAGTGAATTATACGCT|GGCTTATAGGGGGAGCTGCACTCAAGGTCAGCCAAGATGCTGGTCAAGGAGTCAATCTCA
                              >chr3:188242080-188242562 - E=3e-273
GCGTCCAGGCTGGAGCGCCTCTCCTCAAGTGTCTTGCCTCCGGGATTCCCCTGTTGGAAGCAACACAGACATGTTAACAAAGGAAATTT

CCCTTGATATCATTATAAGTTCAAATTTCTCAAGTTATAATGTGGCTTTGTCCAAGTTTTTCTGCACCTCTTTTTTTTTAATTAAGCTT

TCATAAAATCTCAGTGAGGTAGGCTGAGATAGAATTATCACTTTATGACAGATATGAAAACTGAAAAATAAAAGATGATTTTTCTCAGG

TCACACACTTATTTAGCAGTTTTGTCGTTGTTGTTGTTTAAAATAAGTATACAAGTAAAGGAAAATAAATGTCTCCATTCCTGCACCTA

AAGAACAGAACCAGCTGAAGAGAGCTCACAAGCACTTGGTGTTTAGACACCACTGCCTAGAATAA
```
